# Bronchoscopic Treatment of Benign Endoluminal Lung Tumors

**DOI:** 10.1155/2019/5269728

**Published:** 2019-02-27

**Authors:** Levent Dalar, Cengiz Ozdemir, Sinem Nedime Sokucu, Halide Nur Urer, Sedat Altin

**Affiliations:** ^1^Bilim University Faculty of Medicine, Department of Pulmonary Medicine, Istanbul, Turkey; ^2^Yedikule Thoracic Diseases and Thoracic Surgery Hospital, Interventional Pulmonology Unit, Istanbul, Turkey; ^3^Yedikule Thoracic Diseases and Thoracic Surgery Hospital, Pathology, Istanbul, Turkey

## Abstract

**Background and Aim:**

Endobronchial benign tumors are a rarely seen clinical entity but may cause significant symptoms. Endobronchial treatment has the potential for relieving symptoms while saving the patient from invasive surgical procedures. No trials have been published that present and compare the various endobronchial treatment modalities for endobronchial benign tumors. The aim of the present study is to define safety and efficacy of endobronchial treatment in patients with benign endobronchial tumors from the point of complications and success rate.

**Methods:**

This study is a retrospective cohort study from a review of medical charts. Eligibility criteria included diagnosis of a benign endobronchial tumor. Our institution's bronchoscopy and pathology database was searched for specific benign tumors, and the results were further detailed based on the endobronchial location.

**Results:**

Forty-four patients with pathologically confirmed benign endobronchial tumors were included. Tumor regression was achieved in all patients with diode laser and argon plasma coagulation in combination with or without cryotherapy and without any major complication. There were no significant differences between the use of either diode laser or of argon plasma coagulation as a modality with immediate effect from the occurrence of residual tissue that needed cryotherapy (*P* > 0.05). There were no major complications. Eight patients had minor complications including minor bleeding (6 patients) and hypertension (2 patients) that were controlled medically. Thirty-one patients (70%) had very good response, and 13 patients (30%) had good response as defined in literature before.

**Conclusion:**

Diode laser and argon plasma coagulation in combination with or without cryotherapy are safe and effective methods for endobronchial treatment of benign endobronchial tumors.

## 1. Introduction

Benign endobronchial tumors are rarely seen lung tumors and include approximately 2–5% of all lung tumors, of which only 6% occur endobronchially [1–4]. However, benign endobronchial tumors may lead to considerable clinical signs and symptoms including hemoptysis, dyspnea, stridor, and wheezing and may also cause postobstructive pneumonias [2, 4].

Because endobronchial tumors may cause airway obstruction regardless of their benign or malignant character, removal of the tumor is the first treatment of choice to alleviate respiratory symptoms and to dilate and maintain the airway. Conventionally, these benign tumors have been managed with surgical resection [5]. However, bronchoscopic management has been experienced as a widely used skill over the last 30 to 40 years. Bronchoscopic techniques to manage benign endobronchial tumors include neodymium-yttrium aluminum garnet (Nd : YAG), diode laser, argon plasma coagulation, and cryotherapy. There is no consensus, and there have been no randomized trials relating to the use and choice of these techniques in the literature because of these tumors' uncommon occurrences. In this study, we present some proposals concerning the efficacy and most appropriate indications for various conditions of bronchial benign tumors, based on an evaluation of routine work-up data in our charts and patient treatments in order to provide guidance from the point of interventional bronchoscopic treatment for benign obstructive lesions.

## 2. Methods

### 2.1. Study Design and Selection of Patients

The present study is a retrospective cohort study in which we retrospectively reviewed all the patients referred to the interventional pulmonology unit for central airway obstruction between January 2004 and April 2011 and included those who were diagnosed as having endobronchial benign tumors and who underwent endobronchial treatment. All the patients with benign endobronchial tumor were included in the study.

Since the study was a restrospective study, ethical approval was not needed. Argon plasma coagulation (40 Watt, blended mode-continuous flow) was performed using a device manufactured by ERBE Elektromedizine GBMH, (Tubingen, Germany). Standardized protocols for appropriate power selections were used in accordance with the manufacturer's recommendations.

Cryotherapy was performed using the ERBOKRYO system (Elektromedizine GBMH, Tübingen, Germany). A diode laser operating at a wavelength in 980 nm with 4 to 25 Watt, pulsed mode (Biolitec, Ceralas D 25; Jena, Germany) was used for the endobronchial treatment.

All the patients were intubated by a rigid bronchoscope (Efer Endoscopy, La Ciotat, France) under general anesthesia using standard techniques, and mechanical debridement was performed when necessary.

### 2.2. Collection of Data and Measurements

Clinical and radiological data were accessed using the hospital electronic database and radiological systems. The primary outcomes of the present study were the response to treatment of the patients and the rate of complications of the endobronchial treatment. Respiratory symptoms were evaluated as improved, worsened, or unchanged. In postinterventional clinic patients' notes, pre- and postinterventional radiological data were evaluated for improvement in the airway lumen. Any complications during or after the endobronchial treatment were recorded. Total procedures for endobronchial treatment, tumor recurrence, and residual rate were also recorded.

The Chi-square test was used to assess the significance of factors influencing the choice of interventional bronchoscopic treatment modality. APC and diode laser was compared from the occurrence of residual tissue by Chi-square analysis. A *P* value of less than 0.05 was accepted as statistically significant.

## 3. Results

### 3.1. Bronchoscopic Findings and Clinical Characteristics of the Patients

A total 44 patients was included in the study. The range of the patients' ages was between 4 and 86 years (mean, 58 years). There were 33 male (75%) and 11 female (25%) patients in the study population. The locations of the benign tumors were as follows: five of them were located in the trachea (13%); 16 of them were in the right main bronchus (41%); 13 of them were in the left main bronchus (33%); and 5 of them were located at multiple sites (13%). The tumors have obstructed or nearly obstructed the lumen where they were located. Luminal obstruction was between 70% and 100%. Tumor sizes were between 2 mm (left upper lobe apicoposterior segment) and 18 mm (trachea). Patients and their disease characteristics are summarized in Table 1. The study patients presented with dyspnea (80%), cough (50%), constitutional symptoms (30%), hemoptysis (15%), and wheezing (5%). The most common benign tumor was a hamartoma (34.1%) in the present study (Table 2). There were no major complications. Eight patients had minor complications including minor bleeding (6 patients) and hypertension (2 patients) that were controlled medically.

### 3.2. Endobronchial Treatment Modalities

Diode laser, argon plasma coagulation, and cryotherapy were used as an endobronchial treatment modality in the study patients with endobronchial benign tumors. The patients in the study population underwent 22 procedures with diode laser, 21 procedures with argon plasma laser coagulation, and 21 procedures with cryoptherapy for airway obstruction. Diode laser treatment was the most frequently used endobronchial treatment modality with 19 patients (43%) in the present study. The other endobronchial treatment modalities, and their percentages are presented in Table 3 for comparison. There were no significant differences between either the use of diode laser or the use of argon plasma coagulation as a modality with immediate effect from the occurrence of residual tissue that needed cryotherapy (*P* > 0.05).

### 3.3. Response to Endobronchial Treatment

**Figure 1:**
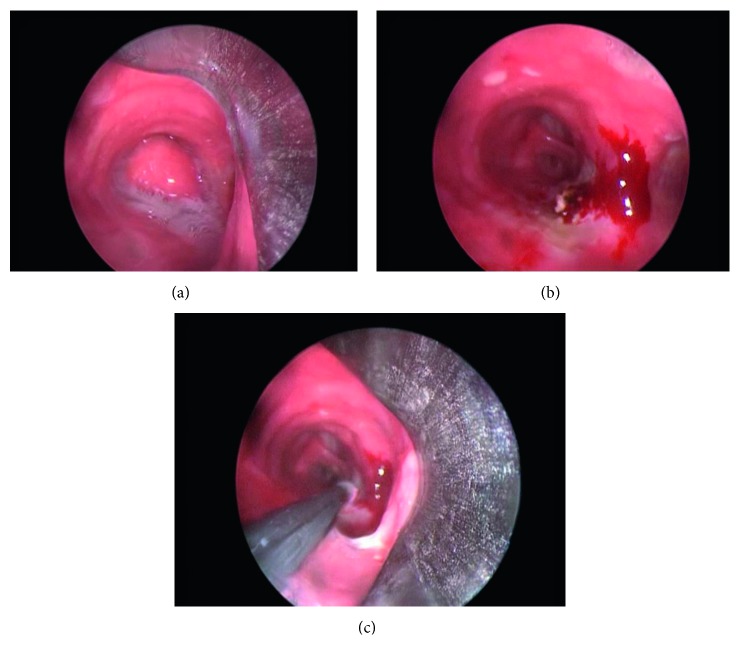
(a) Typical bronchoscopic findings of endobronchial hamartoma with smooth surface and rounded mass having a resemblance to normal mucosa (grade I). (b) Bronchoscopic appearance after core-out of the lesion. (c) Application of cryotherapy for the residual lesion.

The response to endobronchial treatment was classified according to the previous study [2] as “very good” if complete removal of the tumor could be achieved at the first session of endobronchial treatment. If partial removal of the tumor could be achieved and repeated sessions were necessary with no complications, the response to endobronchial treatment was classified as “good”. A “poor” response signified that the lesion could not be removed with the endobronchial treatment. Thirty-one patients (70%) had very good response, and 13 patients (30%) had good response. In this study, no poor response was detected. A sample for very good response can be seen in Figures 1(a)–1(c). There were no major complications in the present study. All of patients reported symptomatic improvement in the study group. Two patients (4.5%) required surgery due to a residual tumor and unavailability of a definitive diagnosis.

### 3.4. Follow-Up

No recurrence was seen in 5 years follow-up period. In first five years, the fibreoptic bronchoscopy was done yearly for relapse. After five years, it has been cautioned that they should be consulted to interventional pulmonology unit in case of any complaints. It has been performed phone visit for this study to state any complaints or sign of recurrence. Authors could reach to 36 patients via phone call, and they have no sign of relapse. The papilloma patient has been pursued for 4 years with any recurrence bronchoscopically; after that time, he was lost to follow-up.

## 4. Discussion

**Figure 2:**
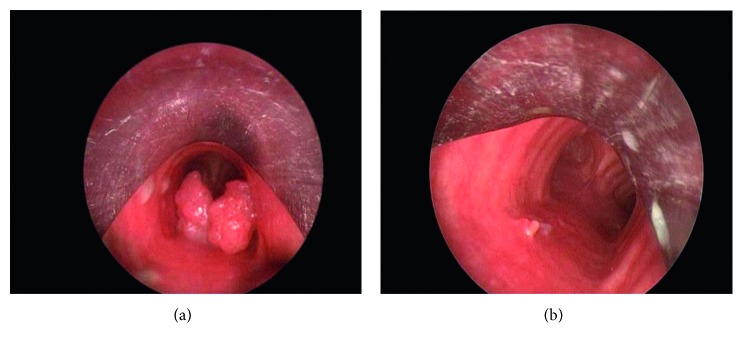
(a) Bronchoscopic appearance of tracheal papilloma. (b) Bronchoscopic appearance 1 month after endobronchial treatment.

The present studies of endobronchial treatment of benign tumors can be classified as nonrandomized trials and observational studies including case series, case reports, and retrospective chart reviews in the literature [4]. There is no consensus statement and no randomized trial about the use and choice of bronchoscopic techniques to manage benign endobronchial tumors in the literature because of the uncommon occurrences of those tumors. No controlled trials have been published that compare the various endobronchial treatment modalities. The majority of patients (95%) with benign endobronchial tumors have been treated using rigid bronchoscopy and have received laser therapy with mechanical resection [2–4, 6]. There are some advantages in using the rigid bronchoscope including the opportunity for the operator to use suction and thus produce a larger amount of debridement, as well as enabling the operator to protect airway patency during the endobronchial treatment. However, flexible bronchoscopy can also be used through an endotracheal tube to enable tumor destruction [4, 7]. All of the endobronchial treatments were performed by rigid bronchoscopy in the present study. Endobronchial treatment through rigid and/or flexible bronchoscopy can be generally divided into two main headings as having either immediate effects or delayed effects. The techniques having immediate effects include laser, thermocoagulation, and argon plasma laser use. The techniques with delayed effects include cryotherapy, photodynamic therapy, and brachytherapy [8]. Immediate effect has a particular importance in a symptomatic patient with an endobronchial benign tumor. In this setting of a symptomatic patient, laser therapy, argon plasma coagulation, and electrocautery are mostly preferred due to their rapid action to destroy tissue for symptomatic relief [7–9]. FiO_2_ must be below 40% during those endobronchial therapies including laser therapy, argon plasma coagulation, and electrocautery due to potential airway fire risks [4, 8]. Bronchoscopic treatment with a laser is most frequently used in benign endobronchial tumors including adenomas, chondromas, lipomas, or schwannomas [2, 7, 8]. Neodymium-yttrium aluminum garnet and diode laser have been used for endobronchial treatment. The diode laser has a much smaller wavelength than the traditional neodymium-yttrium aluminum garnet laser [9]. Diode lasers were most frequently used in the present study as an endobronchial treatment modality, that is, for 19 patients (43%). Shah et al. reported that 62% of benign tumors can be cured by bronchoscopic treatment with a laser [2]. Historically, the best indications for endobronchial laser therapy include the following: a tumors' close endobronchial nature, a limited extension of the tumor within the endobronchial lumen, having a lower potential for recurrence rate, the patient being a poor candidate for surgical intervention, and those symptomatic patients or near symptomatic patients who have a potential risk of airway compromise [2, 7, 8]. The general success rate for endobronchial benign tumors treated by laser has been reported as ranging from 50% to 80% [8, 10]. For papillomas (Figure 2(a)), repeated endobronchial laser treatment is usually needed due to the high frequency of relapse of papillomas (Figure 2(b)) [2, 8] (Fi). Thus, repeated endobronchial therapy was performed for one tracheobronchial papillomatosis case in the present study. Stent application may be beneficial for severe endobronchial papillomatosis [11]. Argon plasma coagulation is an effective and safe method for devitalization of an endobronchial tumor with a rapid onset of action by shrinking the endobronchial tumor. This is rarely described in the literature as a means for treating endobronchial benign tumors. Miller et al. showed good tumor regression using argon plasma coagulation, electrocautery, and cryotherapy either singly or in combination in one case series [12]. Argon plasma coagulation was used with 16 patients (36%) as a good and safe endobronchial treatment modality in the present study.

**Figure 3:**
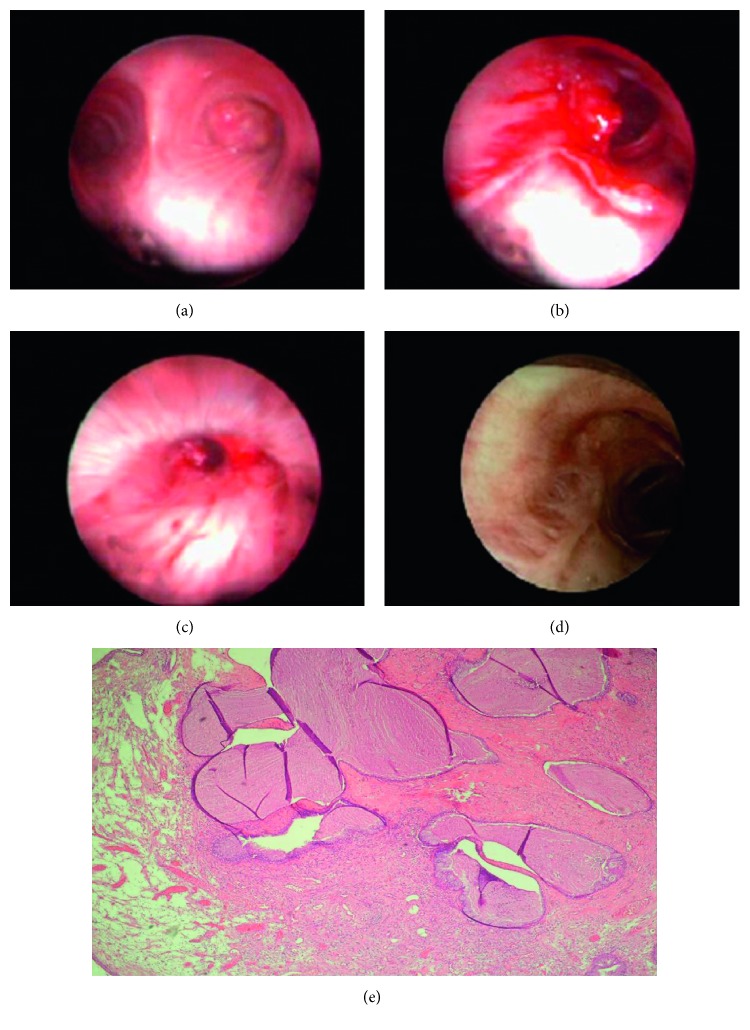
(a) Bronchoscopic appearance of endobronchial mucous adenoma. (b) Bronchoscopic appearance of the tumor during endobronchial treatment. (c) Bronchoscopic appearance 1 month after endobronchial treatment. (d) Bronchoscopic appearance 1 year after endobronchial treatment. (e) Numerous mucus-filled glandular structures throughout the edematous stroma beneath the bronchial epithelium (HE, ×100).

**Figure 4:**
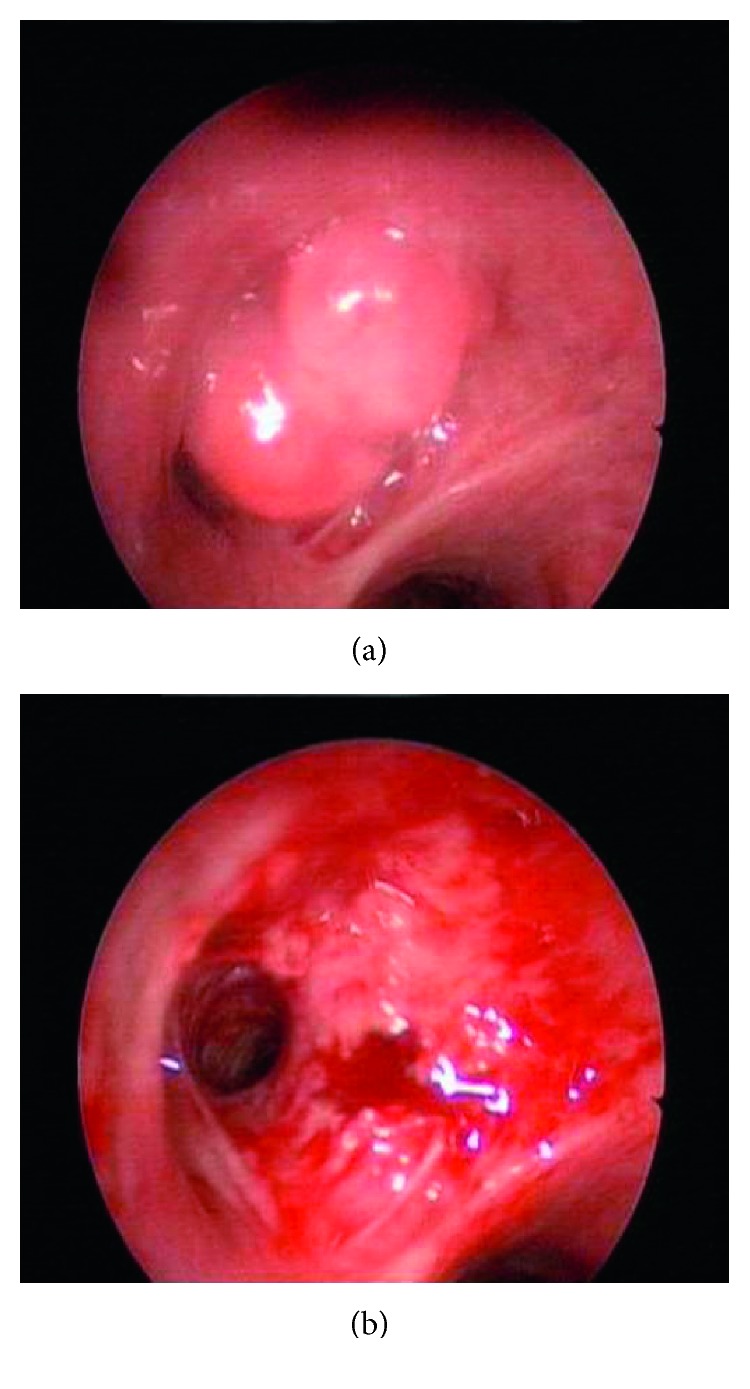
(a) Bronchoscopic appearance of fibroepithelial tumor obstructing left upper lobe superior segment. (b) Bronchoscopic appearance after endobronchial treatment.

Endobronchial cryotherapy has been proposed to be less effective as a single therapy of endobronchial benign tumors [4]. Endobronchial cryotherapy induces tissue destruction by the formation of ice crystals in the cytosol, leading to hypertonicity and extraction of intracellular water [13, 14]. Repeated cryotherapy applications may be necessary for the total removal of the lesion, and this can be classified as main disadvantage of endobronchial cryotherapy. Cryotherapy was used in 13 patients (30%) in the present study, and six cases needed repeated endobronchial cryotherapy for removal of residual tissue for a total cure. Also, cryotherapy is not very useful in emergency settings, because of its relatively slow mechanism of action [7, 15]. Therefore, endobronchial cryotherapy can be applied as a complimentary modality with other endobronchial therapies in the management of benign endobronchial tumors [13, 14]. We used endobronchial treatments having immediate effects including diode laser and/or argon plasma coagulation with or without concurrent cryotherapy. This approach has been summarized in Figures 3(a)–3(e). Endobronchial cryotherapy has been shown to be effective in the management of endobronchial benign tumors in one study and was proposed as a safe method in the management of benign endobronchial lesions and easy to perform [14]. Cryotherapy was used as a single endobronchial treatment modality in the present study in 4 patients diagnosed as having a hamartoma (*n*=3) or a fibroepithelial polyp (*n*=1) (Figures 4(a) and 4(b)) without any recurrence during the follow-up periods. Therefore, endobronchial cryotherapy may be an effective and safe alternative treatment in the management of at least some selected endobronchial benign tumors. The effect of cryotherapy on the rate of recurrence of endobronchial benign tumor is another obscure issue that should be studied in controlled prospective randomized studies. There is no consensus, and a randomized trial about the choice of endobronchial treatment techniques for the endobronchial tumors is needed, particularly endobronchial benign lesions. Therefore, the choice of endobronchial treatment technique used is more often related to the local availability of the modality, the operator's experience, and preference. Indications for the choice of endobronchial treatment in benign endobronchial tumors are not well defined, and in practice, a multimodal approach is often employed and this as in the present study. We found that there were no significant differences between the use of either diode laser or use of argon plasma coagulation as a modality with immediate effect on the residual tissue that needed cryotherapy. Overall, recurrence rates have been reported as 8.4% for endobronchial benign tumors treated bronchoscopically and only 5% of the endobronchial benign tumors require surgery for residual or recurrent tumor [4]. Two patients (4.5%) required surgery in the present study due to a residual tumor and unavailability of a definitive diagnosis. In the present study, all of the patients were cured with endobronchial treatment during the 18 months of the follow-up period. Also, there were no recurrences during follow-up periods of study population in the present study. Pneumothorax, pneumomediastinum, mediastinal emphysemas, and bronchial lymph fistula are rare complications of bronchoscopic treatment of endobronchial benign tumors, reported as 0.9%, 1.4%, 1%, and 0.5%, respectively [2, 4]. Complication rates were similar in the literature and in the present study. There were no major complications in the present study. The mortality rate of endobronchial treatment of benign tumors has been reported as 0.5% [4]. There was no death related to the endobronchial treatment in the present study. Endobronchial treatment should not be an option in patients with total obstruction of the tracheobronchial lumen with total lung or lobar atelectasis. In those patients, surgical resection could be an option instead of endobronchial treatment. The other common indications for a surgical approach to endobronchial lesions are tumor extensions beyond the endobronchial lumen and a tumor's malignancy potential [4, 16, 17].

The incidence of endobronchial benign tumors particularly in the central airways is low. Endobronchial treatment is an available option for those patients with endobronchial benign tumors but when the possibility of malignancy cannot be excluded, or when there are indications for surgical resection including peripherally located pulmonary organizing pneumonia or bronchiectasis secondary to repeated infection, surgical resection of the lung with the including bronchial tumor, is performed in most of the cases [2, 18]. Kajiwara et al. reported their experiences from the point of complication and recurrence rate for interventional management of benign airway tumors in relation to location, size, character, and morphology and found no complications or recurrences [18]. Rodrigues et al. also reported a case series for minimally invasive bronchoscopic resection of benign tumors of the bronchi and concluded that minimally invasive bronchoscopic resection is a safe, effective method for treating selected benign tumors of the main airway [19].

Limitations of this study include that this is not a randomized controlled prospective cohort study that is why there is no control group. It would be better to compare patients who underwent surgical procedures for benign endobronchial treatment with patients who underwent only endobronchial treatment for the future studies.

## 5. Conclusion

Endobronchial treatment of benign tumors is an effective and safe method that could protect symptomatic patients from an unnecessary invasive surgical procedure. There is a need for randomized prospective studies to define the modality that is most useful for the treatment of endobronchial lesions, particularly for benign endobronchial tumors. Endobronchial treatment having immediate effects with endobronchial debulking concurrently used with cryotherapy may offer an effective method for removing benign endobronchial tumors without any recurrence.

## Figures and Tables

**Table 1:** Baseline characteristics of patients who had endobronchial treatment.

Characteristics	Values
Age	
Mean	59
Range	4–86

Male	33 (75%)

Lesion type	
Hamartoma	15 (34.1%)
Hamartochondroma	5 (11.4%)
Hemangioma	1 (2.3%)
Mucous gland adenoma	1 (2.3%)
Fibroepithelial polyp	12 (27.3%)
Papilloma	3 (6.8%)
Lipoma	1 (2.3%)
Inflammatory pseudopolyp	2 (4.5%)
Amyloidosis	4 (9.1%)

Location of endobronchial lesion	
Trachea	5 (11%)
Right main bronchus	16 (37%)
Left main bronchus	13 (30%)
Multiple sites location	5 (11%)
Lobar bronchi	5 (11%)

**Table 2:** Summary of treatment modalities applied to patients with endobronchial benign tumors.

Diagnosis	Age years (mean)	Gender (male : female)	Number of APC/diode laser/cryo procedures	Additional treatment modalities (*n*)
Hamartoma (*n* = 15)	55	12 : 3	5/8/12	Mechanical debridement (2)
Hamartochondroma (*n* = 5)	64	3 : 2	2/2/4	Mechanical debridement (2)
Hemangioma (*n* = 1)	45	1 : 0	—/1/—	—
Adenoma (*n* = 1)	37	1 : 0	1/—/2	Mechanical debridement (1)
Fibroepithelial polyp (*n* = 12)	58	10 : 2	4/5/3	—
Papilloma (*n* = 3)	55	2 : 1	3/2/—	Mechanical debridement (1)
Lipoma (*n* = 1)	64	1 : 0	3/—/—	—
Inflammatory pseudopolyp (*n* = 2)	62	2 : 0	1/4/—	—
Amyloidosis (*n* = 4)	71	1 : 3	3/—/—	Mechanical debridement (2)

APC, Argon plasma coagulation.

**Table 3:** Endobronchial treatment modalities of patients with endobronchial benign tumors.

Endobronchial treatment modality	Total number of procedures	Number of patients (%)
Diode laser	22	19 (43%)
Argon plasma coagulation	21	16 (36%)
Cryotherapy	21	13 (30%)

Cryotherapy was combined in some cases with immediate effect hot endobronchial treatment modalities.

## Data Availability

The data used to support the findings of this study are available from the corresponding author upon request.
